# Nitrogen Isotopes Reveal High NO_x_ Emissions from Arid Agricultural Soils in the Salton Sea Air Basin

**DOI:** 10.21203/rs.3.rs-4249148/v1

**Published:** 2024-04-19

**Authors:** Heather C. Lieb, Matthew Maldonado, Edgar Ruiz, Christian Torres, Luis Olmedo, Wendell W. Walters, Ian C. Faloona

**Affiliations:** 1. Department of Land, Air, and Water Resources, University of California, Davis; 2. Comité Cívico del Valle; 3. Department of Chemistry and Biochemistry, University of South Carolina

## Abstract

Air quality management commonly aims to mitigate emissions of oxides of nitrogen (NO_x_) from combustion, reducing ozone and particulate matter pollution. Despite such efforts, regulations have recently proven ineffective in rural areas like the Salton Sea Air Basin of Southern California, which routinely violates air quality standards. With $2 billion in annual agricultural sales and low population density, air quality in the region is likely influenced by year-round farming. We conducted NO_x_ source apportionment using nitrogen stable isotopes of ambient NO_2_, which indicate a substantial contribution of soil-emitted NO_x_. The soil source strength was estimated based on the mean δ^15^N-NO_x_ from each emission category in the California Air Resources Board’s NO_x_ inventory. Our annual average soil emission estimate for the air basin was 11.4 ± 4 tons/d, representing ~30% of the extant NO_x_ inventory, 10× larger than the state’s inventory. Therefore, the impact of soil NO_x_ in agricultural regions must be re-evaluated.

## 1 Introduction

Nitrogen oxides (NO_x_ = NO + NO_2_) are a precursor to ozone (O_3_) and particulate matter (PM), both of which are federally regulated criteria pollutants, known to negatively impact human health and the environment.^1^ NO_x_ is produced through natural, biogenic, and anthropogenic processes, although a critical distinction is necessary. Natural sources of NO_x_ occur without influence from human activity, such as lightning or weather-induced wildfires. Biogenic NO_x_ is naturally occurring and is produced by living organisms through microbial nitrification/denitrification cycles in soils. Further, anthropogenic NO_x_ results from human activities such as fossil fuel combustion in vehicles and power plants. However, these natural processes can be affected by anthropogenic activity, such as biomass burning in agricultural systems, human-ignited wildfires in poorly managed landscapes, and microbial activity in soils of fertilized croplands.

**Figure 1.**
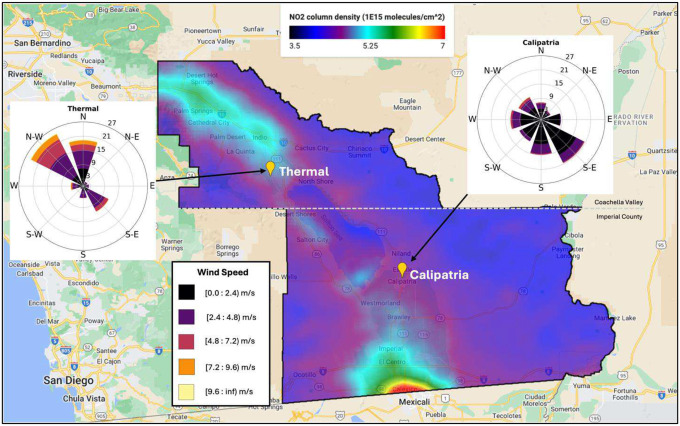
NO_2_ column density was averaged from June 1, 2022, to May 31, 2023, from TROPOMI satellite data for the Salton Sea Air Basin to show the average spatial variation of NO_2_. Calipatria (SE) and Thermal (NW), our two sampling sites, are indicated by a yellow marker. Wind roses for Calipatria and Thermal are also shown.

Anthropogenic combustion sources have traditionally been the primary focus of air quality management practices, as they have historically been the dominant source of NO_x_ emissions, with biogenic processes accounting for about 20% of the global NO_x_ budget, on average.^2–8^ These practices have largely reduced precursor emissions, yet several of the United States’ worst-air quality districts remain in rural regions,^9,10^ including the Imperial and Coachella Valleys of Southern California. These valleys together constitute the Salton Sea Air Basin (SSAB, Fig. 1), which is currently nonattainment for the O_3_, PM_2.5_, and PM_10_ National Ambient Air Quality Standards (NAAQS). Interregional transport from larger metropolitan areas is often blamed,^11–16^ hewing to the traditional paradigm of combustion dominant sources. This issue is a serious matter of environmental injustice because residents of Imperial County are 86% Latino and 21% of residents live below the poverty line.^17^ Additional burdens these communities face include insufficient housing availability and poor infrastructure, language barriers, asthma, and impaired water quality.^18,19^ These factors put residents at risk of respiratory damage and diseases like asthma and COVID-19.^20^ In fact, Imperial County held both the highest infection and mortality rates per capita in California from COVID-19 throughout the height of the pandemic.^21,22^ Members of the rural Eastern Coachella Valley also suffer from similar socioeconomic and environmental injustices.^19^

The SSAB is an agriculturally active desert responsible for over $2 billion in annual agricultural sales due to mild temperatures and abundant sunshine in the winter, allowing for year-round production.^23^ However, growing crops in sandy soil is challenging and can require anthropogenic land modifications such as carbon-based amendments, heavy application of inorganic fertilizers, and intensive irrigation. This is primarily due to the soil’s texture, with larger particle diameters and lower organic carbon composition, resulting in poor retainment of water and nutrients necessary for plant growth.^24^ Tillage is often required for agricultural production in sandy soils, which can result in structural damage to the soil, leading to compacted soils that are difficult to cultivate when dry, as well as entrainment of particulate matter.^24–26^ Hard-setting soils have high soil strength that is not suitable for root growth or shoot development. Consequently, this requires frequent soil-wetting through irrigation practices, especially in summer months when temperatures regularly exceed 35°C; these dry, hot conditions and changes in volumetric soil moisture (VSM) may lead to large, transient soil NO_x_ emissions (Figs. 1 & S1).^10,24,27^

In addition, inorganic fertilizer amendments are not regulated, leading to over-application and nutrient leaching into the surrounding environment, such as the groundwater, local water sources, atmosphere, and soils.^24,28^ The objective of this work is to understand the implications of agricultural practices in arid agroecosystems of the SSAB on regional air quality. Observations and modeling studies show a massive expansion of arid lands globally due to climate change, ranging from 2.5 – 6.5 million km^2^ by the end of the century, especially in large croplands in the northern hemisphere.^29^ Consequently, agricultural production in arid ecosystems will become more prominent,^30^ therefore the implications of these practices need to be better understood. Microbes in soils naturally produce NO and nitrous oxide (N_2_O) gases (and nitrous acid gas, HONO) by the same processes, but their relative amounts are highly uncertain. It is believed that they are produced as intermediates in the nitrification of ammonium to nitrate and anaerobic denitrification of nitrate to N_2_, but the proportions that emerge as NO and HONO depend on soil conditions like nutrient availability, soil moisture, and temperature.^31–34^ In dry, well-aerated soils like in the Salton Sea Air Basin, nitrification is likely the dominant process resulting in soil NO emissions, which can diffuse out of the soil before being consumed in further biogeochemical cycling. However, in wet soils, denitrification dominates, and NO is mostly consumed before leaving the soil, resulting in pulses of N_2_O. In soils that are more wet and highly anaerobic, N_2_O is further reduced to N_2_ (Firestone & Davidson, 1989). Soil NO emissions are believed to increase exponentially with temperature, then plateau between 30–40°C, as long as nutrient availability and soil moisture are not limiting.^34–36^

Soil pH is noteworthy because high pH generally factors microbial ecosystems that promote nitrification and NO production, while acidic fertilized soils produce HONO through chemical equilibrium with nitrite in water.^34^ However, Oswald et al. (2013) found comparable quantities of HONO and NO emitted from nonacidic soils, suggesting that HONO may be an unaccounted, yet significant amount of reactive nitrogen released from soil worldwide, particularly dominant in arid and arable environments like the SSAB. This is crucial as the daytime lifetime of HONO with respect to photolysis of NO + OH is around 15 minutes, causing soil emissions to rapidly cycle into the photostationary state of O_3_-NO-NO_2_, becoming an additional source of NO_x_ to the atmosphere.^37^

As NO_x_ emissions from combustion sources decline under ongoing air quality regulations, it becomes crucial to comprehend the impact of anthropogenically induced biogenic NO_x_ production, particularly from agricultural soils. The nitrogen stable isotope composition (δ^15^N) is proposed as a valuable tool for NO_x_ source apportionment due to differences in mean δ^15^N-NO_x_ values between anthropogenic and biogenic emission sources.^38–40^ Ambient δ^15^N-NO_2_ is recognized as an effective means of source apportionment, benefiting from well-quantified isotope fractionation factors and offsetting isotope effects resulting from the interplay between isotope equilibrium and photochemical fractionation^41^ Previous ambient δ^15^N-NO_2_ studies have successfully tracked the influence of highly variable biogenic soil NO emissions impacting the NO_x_ budget in a small Midwestern city during the summer,^42^ suggesting it could be a powerful tool for constraining biogenic soil emissions in the SSAB.

In this study, δ^15^N was quantified from NO_2_ (and total nitrate, tNO_3_) actively collected on a Thermo Scientific^™^ ChemComb^®^ Speciation Cartridge (CCSC). Ambient NO_2_ samples were collected in two locations in the SSAB for 10 months and δ^15^N was analyzed to quantify the soil source contributions to the region’s NO_x_ inventories. Using our field measured δ^15^N-NO_2_ values and accounting for nitrogen isotopic fractionation to adjust these values to δ^15^N-NO_x_, we used a mixing model approach to estimate the soil source strength of the field observations based on the mean δ^15^N-NO_x_ from each emission source and from the *a priori* source apportionment reported in CARB’s California Emissions Projection Analysis Model (CEPAM) inventory. Because nitrate is influenced by long-range transport, we do not use tNO_3_ as a marker for source apportionment. This study aims to improve our understanding of the sources of persistent air pollution in the SSAB by assessing the current NO_x_ inventory’s estimation of soil NO_x_ emissions.

## 2 Methods

### 2.1 Sample Collection, Extraction, and Isotopic Analysis

Ambient NO_2_ and total particulate nitrate (tNO_3_) were collected and quantitatively speciated using a denuder-filter pack active sampling device known as a ChemComb Speciation Cartridge (CCSC), as previously described.^43^ Briefly, the body of the CCSC contains an inlet with an impactor plate for the removal of coarse particles, a glass transition piece to induce laminar flow, two honeycomb denuders to collect ambient NO_2_ gas, and a downstream filter pack.^44^ Denuders were soaked in 10% (v/v) hydrochloric acid (HCl) for 24 hours, then rinsed in triplicate with 18.2 MΩ Millipore water and air-dried prior to field preparation. For the collection of NO_2_, denuders were coated with 10-mL of 2.5 M potassium hydroxide (KOH) and 25% by weight of guaiacol (C_7_H_8_O_2_) using methanol as a solvent, which was allowed to air dry, then capped until assembly for field analysis.^45–47^ This mixture selectively binds NO_2_ as nitrite (NO_2_^−^). Following the denuders is a single Nylon filter (Measurement Technologies Laboratory NY47P, 47 mm and 1 μm pore size) for the collection of tNO_3_. Ambient air was sampled at a flow rate of 10 LPM using a mass-flow controller (MKS IE50A008304SBVP20) and vacuum pump. Details about chemicals and materials used can be found in Supplementary Table S1. Field sampling was performed from June 2022 – April 2023 at two sampling sites in the SSAB (Fig. 1), one in the Imperial Valley (Calipatria High School in Calipatria, CA) and one in the Coachella Valley (Torres-Martinez air station in Thermal, CA). This project incorporated community engagement and citizen science; Comité Cívico del Valle (CCV), a grassroots community organization based in Imperial County that was founded on the principle that “Informed People Build Healthy Communities”, served as our community partners. Trained air monitoring technicians from CCV oversaw field set-up and break-down. Sampling was performed for 3–7 days, approximately monthly over a period of ten months.

The extraction of NO_2_ and tNO_3_ was performed with 30-mL and 20-mL of MQ water for the front denuder and nylon filter, respectively, then vacuum filtered through a Whatman grade 1 filter. Back denuders were used to check for breakthrough and were not used for isotopic analysis. Field blanks were collected for method validation, however, blanks contained negligible concentrations of NO_2_ and tNO_3_. Nitrate (NO_3_^−^) and nitrite concentrations in aqueous solution were determined on a UV-Vis spectrophotometer using a colorimetric method developed by Doane and Horwath (2003) (extraction methods in Text S2).^48^ NO_2_ denuder extracts were neutralized with 0.1 M HCl, then isotopic analysis was performed at the UC Davis Stable Isotope Facility by the denitrifier method on a GasBench-PreCon-Isotopic Ratio Mass Spectrometer to determine δ^15^N-NO_2_.^49,50^ The pooled standard deviation for the ^15^N reference was 0.11‰.

The lifetime of aerosols in the atmosphere is typically 5–10 days, meaning the N in particulate nitrate is derived from a large integrated area that spans several air basins across California. Although there was fair correlation between NO_2_ and particulate NO_3_ δ^15^N values across the experiment (Pearson correlation coefficient of ~0.45), the average for the NO_3_ samples was ~10 ‰ heavier than for NO_2_, indicating that the aerosol nitrate was likely more strongly influenced by distant, traditional fossil-fuel combustion sources of NO_x_. Additionally, quantifying isotope effects associated with tNO_3_ formation is more difficult.^51^ Uncertainty in knowing exactly where the tNO_3_ was produced along its long-range trajectory makes it a much more challenging marker for source apportionment studies, therefore we do not use the tNO_3_ data (but it is reported in Table 3).

### 2.2 Evaluation of δ^15^N References

The isotopic signature of nitrogen is expressed in terms of its δ^15^N, where:

$$\delta^{15}N=\left(R_{\text{sample}}/R_{\text{standard}}-1\right)\times 10^{3}$$

In this equation, R represents the ^15^N/^14^N ratio. The standard is atmospheric N_2_, which is considered to have a globally uniform isotopic composition.^52^ Studies of the natural variations in ^15^N/^14^N ratios from different sources are used to apportion sources of NO_x_ pollution.

A literature review was conducted to evaluate published stable ^15^N isotope ratios for various sources of NO_x_, and relevant δ^15^N-NO_x_ values are summarized in Table 1. On average, biogenic soil sources have been observed to have a significantly larger ratio of light ^14^N isotopes than combustion sources. This phenomenon can be elucidated through the “leaky pipe” analogy, a concept derived from the hole-in-the-pipe model proposed by Davidson and Firestone.^31^ In the “leaky pipe” scenario, soil NO-producing processes, such as nitrification, denitrification, and chemodenitrification, act as microbial conduits where microbes preferentially utilize the lighter ^14^N isotope. This preference arises from kinetic factors that favor the incorporation of the lighter N isotope, leading to a more depleted δ^15^N signature.^45,52,53^ The “leaky pipe” effect in soils contributes to the observed larger variations reflected in the standard deviations reported in the literature (Table 1 and Supplementary Fig. S4).

It is important to consider the uncertainties of the isotopic signatures for each source due to variations in measurement techniques found in the literature. For this study, passive sampling techniques^54–56^ were excluded because the measurement periods were much longer, could allow for a mixture of NO_x_ sources, and are more difficult to reproduce, ultimately introducing a bias in the δ^15^N-NO_x_.^57^ Additionally, studies on vehicles without catalytic converters were excluded^58^ since catalytic converters are mandatory and regulated through California smog checks. For these reasons, only two publications were used to represent mobile source emissions: Miller et al. (2017) and Walters, Goodwin et al. (2015).^59,60^ Not all measured values reported in Walters, Goodwin et al. (2015) were used because some measurements were taken from engines that were cold and/or in neutral. Catalytic converters take a few minutes to warm up and work efficiently, and efficient catalytic converters preferentially remove lighter ^14^N isotopes,^59,60^ leading to heavier ^15^N emissions. Therefore, cold engines are biased with more negative isotopic signatures and likely do not represent background mobile source emissions measured in the Salton Sea Air Basin. Additionally, both studies were performed in the northeastern US in large metropolitan areas where biogenic sources are believed to be negligible. The reported mean from Miller et al. (2017) was then averaged with the mean of warm and/or driven vehicles from Walters, Goodwin et al. (2015) to represent mobile source δ^15^N-NO_x_ (Table 2). Further, coal burning is not a method of energy production in the Salton Sea Air Basin or surrounding areas, therefore, studies that measured the isotopic signature of NO_x_ produced through coal combustion have been excluded from our stationary source measurements.^58,61^ See Section 2.3 for uncertainty propagation.

### 2.3 Soil Source Strength Mixing Model Estimation and Propagation of Uncertainty

A best estimate of the sources of NO_x_ in the SSAB is compiled in the California Air Resources Board’s California Emissions Projection Analysis Model (CEPAM) shown in Table 2. CEPAM was used over the EPA’s National Emissions Inventory (NEI) because CEPAM is broken into county and air basin, and due to its California focus, should in principle be more accurate. Because chemical and physical processing can induce isotopic fractionation such that δ^15^N may not be conserved, isotopic fractionation was calculated to determine δ^15^N-NO_x_ (see Text S3 and Eq. S1–3).^51,62^ These δ^15^N-NO_x_ values were then used to calculate the soil source emission strength (*E*_*s*_) in Equation 1 based on the average δ^15^N-NO_x_ of each emission source based on the *a priori* source apportionment reported in the CEPAM NO_x_ inventory, which is used for regulatory modeling in California. Here, *δ*_*obs*_ represents the observed δ^15^N-NO_x_, *i* represents the main source types accounted for in the CEPAM NO_x_ inventory (where *a, b, c,* and *s* represent mobile, biomass burning, stationary, and soil sources, respectively), *α*_*i*_ represents the proportion each source contributes to the overall CEPAM NO_x_ inventory, *δ*_*i*_ represents the literature derived δ^15^N-NO_x_ for each source (Table 2), and *E*_*inv*_ represents the total NO_x_ budgeted in the CEPAM inventory (in tons/d).

(1) Es=(δobs−∑a,b,c,siαiδi)Einv(δs−δobs)

Equation1 assumes that the current CEPAM inventory is correct aside from the soil source because the observed δ^15^N was (usually) lighter than expected; without adjusting the soil source signature, the CEPAM inventory would indicate a mean δ^15^N-NO_x_ of −5.04‰ and −3.11‰ for Imperial County and the Coachella Valley, respectively. Therefore, we solve for the magnitude of the soil source such that the observed δ^15^N signature can be explained by the sum of the inventory sources plus the *revised* soil emissions. This procedure further assumes that the sampled NO_2_ is entirely from NO_x_ (or HONO) emitted from sources within the air basin. Figure 1 shows annual NO_2_ column data from the European Space Agency’s TROPOspheric Monitoring Instrument (TROPOMI) for the year of our sampling.

We propagate the various uncertainties associated with these measurements of sources from the literature to estimate the overall error associated with our calculated soil emissions. Equation 2 comes from the propagation of error for the final soil source strength $\left(\sigma_{E_{s}}\right)$. The analytical uncertainty δ_obs_ (on average 0.11‰) was ignored because it is an order of magnitude smaller than the standard deviations of the δ^15^N-NO_x_ measurements for each source (Table 1).

(2) $$\sigma_{E_{s}}^{2}=\sum_{a,b,c}^{x}\frac{\alpha_{i}^{2}E_{inv}^{2}}{{\left(\delta_{s}-\delta_{obs}\right)}^{2}}\times \sigma_{i}^{2}+\left[\frac{\alpha_{i}^{2}E_{inv}^{2}}{{\left(\delta_{s}-\delta_{obs}\right)}^{2}}-\frac{2\left(N-\alpha_{s}^{2}\delta_{s}E_{inv}^{2}\right)}{{\left(\delta_{s}-\delta_{obs}\right)}^{3}}+\frac{N^{2}+\alpha_{s}^{2}\delta_{s}^{2}E_{inv}^{2}}{{\left(\delta_{s}-\delta_{obs}\right)}^{4}}\times \sigma_{s}^{2}\right]$$

where $N=\left(\delta_{obs}-\sum_{a,b,c,s}^{i}\alpha_{i}\delta_{i}\right)E_{inv}$

### 3.1 Results and Discussion

**Figure 2.**
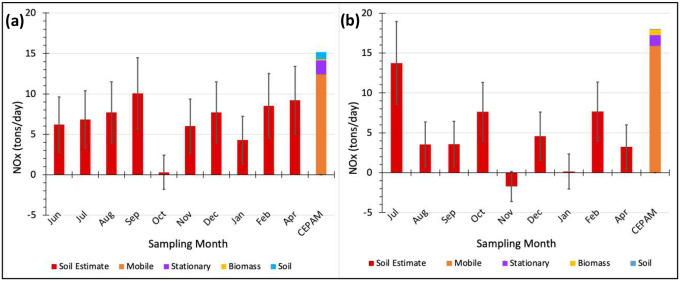
The NO_x_ budget (in tons/day) based on CEPAM2019v1.03 for the year 2022 is shown, which is split into mobile, stationary, biomass burning, and soil emission sources for (a) Calipatria and (B) Thermal. Soil emission estimates from this study are shown in red, and estimated uncertainties are denoted by the error bars. The annual total CEPAM NO_x_ inventory for Imperial County (A) and the Coachella Valley (b) is presented on the right, where mobile, stationary, biomass, and soil sources are shown in orange, purple, yellow, and blue. Note that in Thermal for the November sampling, there was a negative estimated soil contribution, but we assume that the soil source was zero for this month and justified this assumption due to the error bar range.

Results of collected δ^15^N-NO_2_ signatures are shown in Table 3. Figure 2 displays the soil emission estimates for this field campaign at both sites, comparing the monthly magnitudes to the overall annual CEPAM NO_x_ inventory. It is apparent that the CEPAM NO_x_ budget underestimates the soil contribution to ambient NO_x_ (and consequently to PM_2.5_ and O_3_) in both Calipatria (Imperial Valley) and Thermal (Coachella Valley). Soil NO_x_ contributed 0.3 – 10.1 (mean: 6.7 ± 3) tons/d and 0 – 13.8 (mean: 4.7 ± 4) tons/d throughout the duration of the field sampling for the Calipatria and Thermal sites, respectively. These soil emissions amount to on average 11.4 ± 4 tons/d throughout the entire SSAB, an order of magnitude larger than what is represented in the inventory (1.0 ton/d).

The quantitative basis of our estimates relies on the assumption that the ambient NO_2_ at the sampling site comes from sources entirely within the SSAB. Figures 1 (and Supplementary Fig. S1) show the overall pattern of column NO_2_ observed by TROPOMI during its overpass time near 13:30 local time. The satellite data illustrates two important aspects of the regional NO_x_. First, although there is a flow connection between the Coachella Valley and upwind urban sources in the LA basin (Supplementary Fig. S2), the valley plume is distinct and does not appear to simply represent a decaying tail (especially noticeable in the Fall and Winter maps of Supplementary Fig. S1). The Imperial Valley, on the other hand, is only downwind of Mexicali during July/August (Supplementary Fig. S3) and yet it exhibits a broad amorphous shape that deviates from the circular symmetry of a concentration field falling off with distance from an urban core. Second, the basin-wide concentrations are greatest during spring/summer when the photochemical lifetime of NO_x_ is shortest, supporting our finding that agricultural soil sources are significant, especially during the warmest part of the growing season. Regardless of the details of source apportionment for the sampling, Equation 1 shows that any influence from urban sources upwind (with their heavier δ^15^N fractions) would effectively increase our soil emission estimates, meaning that the values reported here represent lower limits. Further details of the regional flow and NO_x_ advection are discussed in Text S1.

### 3.2 Environmental Influences on Soil NO_x_ Production

**Figure 3.**
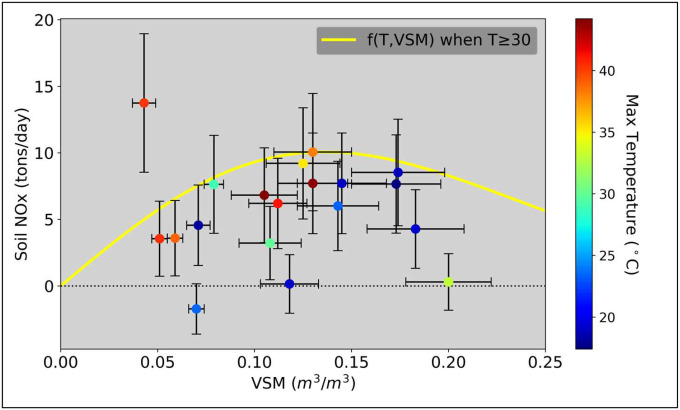
Soil NOx estimates from our field campaign compared to the average VSM and maximum temperature during the measurement period. Maximum temperature was determined for the sampling period from the CARB meteorology station at Calipatria and Thermal. VSM was determined for the Imperial Valley for Calipatria measurements and the Coachella Valley for Thermal measurements using SMAP satellite data, filtered for 4:30 pm (closest time to when T_max_ occurs).

To better understand the environmental influences that control the production of soil NO_x_, we look at the leading parameters used in modeling estimates; namely nutrient availability, temperature, and soil moisture.^36,63^ Temperature is known to strongly influence soil NO_x_ production, although the exact dependence at higher temperatures is still debated,^35^ and has been observed by satellite over croplands.^27^ Nevertheless, no clear temperature dependence was observed in this study (Figs. 2 & 3) despite observations ranging from 17–44°C (Supplementary Tables S3, S4, and Fig. S6). As an agriculturally active desert, NO_x_ production is likely limited not principally by temperature as in unmanaged landscapes but by nutrient abundance and soil moisture determined by agricultural activity.

**Figure 4.**
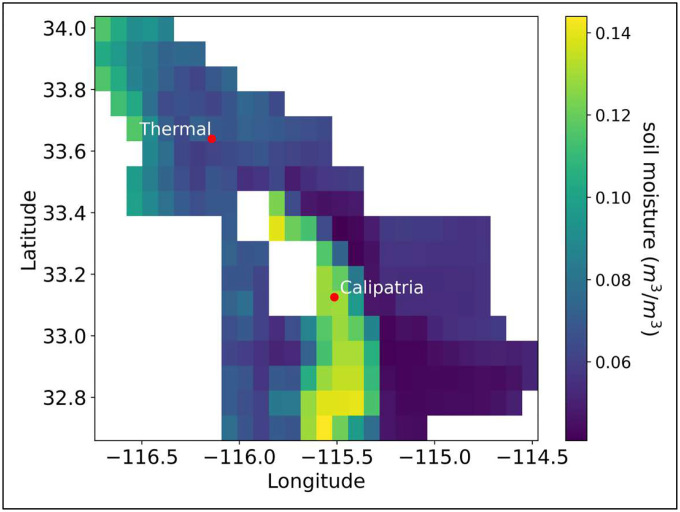
Average volumetric soil moisture (VSM) for the SSAB from June 2022 – May 2023, obtained from Soil Moisture Active-Passive (SMAP) satellite data.

To further understand the influence of these parameters, we looked at volumetric soil moisture content using NASA’s Soil Moisture Active Passive (SMAP) satellite data. Soil moisture tends to be higher on average in the Imperial Valley agricultural lands than in any other region in the air basin (Fig. 4). This is because of the region’s appropriation of approximately 3 billion m^3^ of water from the Colorado River, used primarily for frequent irrigation of its ~200,000 ha of arable soil. It is equivalent to ~150 cm of water over the croplands whereas the climatological rainfall observed usually only amounts to < 5–10 cm (Supplementary Tables S5 and S6).

**Figure 5.**
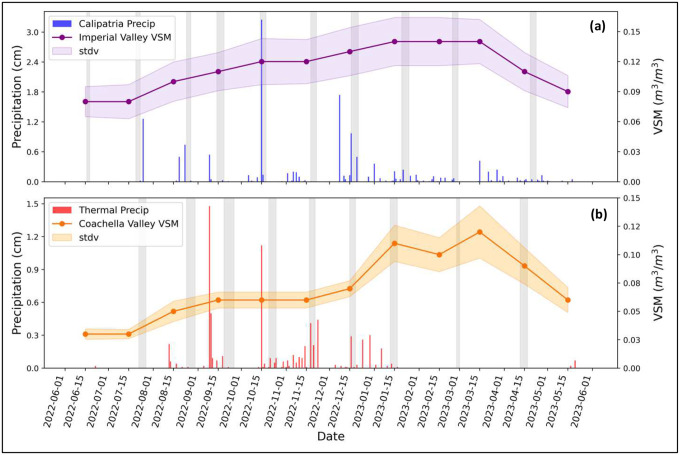
Monthly average Volumetric Soil Moisture, VSM (2022–2023, shown as a line plot), and the standard deviation (shown with shading) in (a) Imperial Valley and (b) Coachella Valley compared to the total daily precipitation (2022–2023, shown as bars) at our field sampling sites. Precipitation data was obtained from AQMIS and CIMIS and VSM data was obtained from NASA’s SMAP satellite. The grey boxes indicate our sampling dates at each site.

Additionally, δ^15^N outliers were observed for October in Calipatria and for November and January in Thermal, yielding estimated soil emissions that were effectively zero within the errors of our measurement technique (Fig. 2). The outliers measured in October (Calipatria) and November (Thermal) correspond to precipitation events during our measurement periods that likely suppressed NO_x_ emissions during the short intervals of sampling by wetting the soil to excess temporarily (Fig. 5).^36^ During our sampling interval in January (Thermal), precipitation was negligible, but examining HYSPLIT back trajectories for the interval, it appeared that there was a high-pressure system over the region that may have forced subsidence of cleaner tropospheric air into the Coachella Valley, which is consistent with the sampled NO_x_ being lower than the monthly average by 5.5 ppb (Supplementary Tables S4 and S6). However, this circulation did not persist throughout the entire 5-day sampling period, and the February sample was also collected when NO_x_ was 5 ppb lower than average, so ultimately, we are uncertain as to the exact cause of this near zero soil emissions measured at Thermal in January.

**Figure 6.**
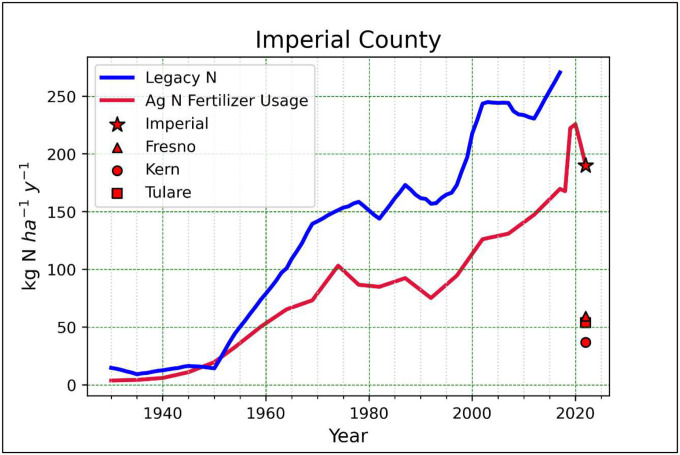
Imperial County N fertilizer usage (red) and legacy N history (blue) was prepared using the Trajectories Nutrient Dataset for Nitrogen (TREND-nitrogen) from Byrnes et al., 2020. The agricultural fertilizer use was extended after 2017 using the CDFA agricultural statistics for N fertilizer sales. Fresno, Kern, and Tulare Counties are ranked 1^st^, 2^nd^, and 3^rd^ in state agricultural sales, while Imperial County is ranked 9^th^. Fertilizer usages are reported as kg of N used per hectare of agricultural land.

Although Figure 3 could be interpreted as exhibiting a crude correspondence between soil NO_x_ emissions and VSM, temperature only appears to be weakly related, which likely indicates that nutrient availability is the dominant factor controlling soil NO_x_ production in this heavily agricultural region. According to the California Department of Food and Agriculture (CDFA), N fertilizer sales in Imperial County have increased by 137% since 1991,^23,64^ and an estimated 18-fold since 1930,^65^ much greater than the national average sevenfold increase. In addition, despite being ranked as the 9^th^ highest in county agricultural sales in the state, Imperial County used more N fertilizer than any other county during 2022 (8% of California total), including the top three crop producing counties (Fig. 6), likely a result of the insufficient nutrient retainment in sandy soils.

Spatially and temporally relevant fertilization data are not currently publicly available, therefore it is difficult to assess the direct influence of this leading parameter on the soil NO_x_ emission variance observed in this study. In Figure 6, we used data from the Trajectories Nutrient Dataset for Nitrogen (TREND-nitrogen) to look at historical N fertilizer applications and legacy N concentrations in Imperial County. N surplus was calculated using TREND-nitrogen, where the county-scale surplus or “legacy” nitrogen (kg N ha^−1^ y^−1^) is calculated by mass balance from the inputs of atmospheric deposition, fertilizer application, biological N fixation, and human N waste, minus the crop and pastureland N uptake.^65^ It is apparent that N fertilizer usage and surplus N in Imperial County has been steadily increasing from 1920 to the present day (Fig. 6). Our estimates are based on a small fraction of days in the Salton Sea Air Basin; in our study, we sampled ~39 days in Calipatria and ~43 days in Thermal, accounting for about 11% of the year. Therefore, our estimates are based on a small fraction of days in the SSAB. Inspecting the conditions of our 19 sampling intervals with respect to the annual values for VSM and air temperature (Supplementary Fig. S5 and Table S7), they compare fairly well.

Nevertheless, without knowing the exact timing of N amendments to all the cropped area, it is quite possible that while our estimates of annual average VSM and temperature are not statistically different from the average, the soil emissions, predominately influenced by the fertilizer inputs, may be much higher than our estimates having missed many periods of high fertilization in our sporadic sampling.

To understand the overall influence of fertilizer inputs on atmospheric N, we calculated the NO_x_ flux as a proportion of fertilizer consumption using the net fertilizer usage recorded.^23^ Imperial County purchased 57,630 tons of all Nitrogen fertilizer, minerals, and compost in 2022.^23^ Our annual average soil emission of 6.7 tons NO_x_/d in Imperial County implies an average flux of 1.3% of the applied N being released as NO or HONO from agricultural fertilizer inputs. This is squarely within the 0.3–2.5% range reported by Jaeglé et al. (2005). Note that we were unable to quantify this for the Coachella Valley because it is part of the much larger Riverside County and fertilizer sales are only reported county-wide.

### 3.3 Comparison Between Studies

To further assess the validity of our results for Imperial Valley, we compared our measurements to Oikawa et al. (2015), which found some of the highest soil NO_x_ emissions on record from chamber studies across the growing season. They further tested their findings by using WRF-Chem to compare against ambient NO_x_ measurements from the air quality network. The default soil emissions in the model needed to be enhanced by an order of magnitude to match their chamber measurements more closely, and the enhancement was found to minimize the model’s root-mean-square error relative to ambient measurements for one week in September 2012. To compare our integrated valley emissions to the modeled surface fluxes, we used the area of agricultural land in the Imperial Valley to be 270,500 ha based on MODIS satellite land surface characterization.^27^ The integrated emissions for their one week in September (the month of our highest estimate of 10.1 ± 4 tons/d) amounts to 17.2 tons/d (Table 4). That study also attempted to match agreement with satellite NO_2_ measurements and found that the emission rate that corresponded best was more like 111.2 tons/day. Their study also found that such soil NO_x_ emission increases led to concomitant rises in surface level ozone of 2–9 ppb, highlighting the importance of this source to air quality and the observed disappearance of ozone abatement in the region.^9^

Almaraz et al. (2018) observed the highest modeled soil NO_x_ emissions published to date for Imperial Valley.^10^ Their method modeled the spatial distribution of soil NO_x_ emissions using an N isotope model in natural areas and an Integrated Model for the Assessment of the Global Environment (IMAGE) in cropland areas to estimate N losses from soils based on surplus N. Their average NO_x_ flux estimated for agricultural lands in Imperial County was ~140 tons/day (51.2 kg N ha^−1^ yr^−1^). Our results were also compared to a modeling study performed using an enhanced version of Fertilizer Emission Scenario Tool for the CMAQ (FEST-C) agroecosystem model paired with the Air Pollution Emission Experiments and Policy Analysis (APEEP), further abbreviated as FEST-C*^66^ Data from their 2017 model for Imperial County indicates an annual average soil emission rate of 13.5 tons/day. Finally, Guo et al. (2020) modeled soil NO_x_ emissions using the DeNitrification-DeComposition (DNDC) model finding the total contribution to the state NO_x_ inventory was 1.1%.^67^ However, they did point out a region of anomalously high emissions in the Imperial Valley, with an annual flux of 0.58 kg N ha^−1^ yr^−1^.

While there is considerable variability between the various estimates presented (Table 4), undoubtedly in part due to the highly episodic and transient nature of soil NO_x_ emissions, the CEPAM inventory estimate is consistently much lower than the other models and observations. We expect that the dominant control of the instantaneous emission rates is the N amendments made to the agricultural soils daily, so the wide range presented above is likely consistent with such inherent variability. Therefore, to obtain an accurate annual average emission rate from soils will take much more comprehensive understanding of N availability in the soil across these highly variable croplands. More work is needed to better constrain the impact of N availability on soil NO_x_ emissions, as well as the precise soil conditions that control microbial activity and gas-exchange. As of 2022, the Sustainable Groundwater Management Act was modified to require farmers in California to report their daily N inputs for consequences of excess N leaching into groundwater. This emerging data set will be crucial for future studies to investigate soil NO_x_ emissions in arid agricultural environments and accurately assess its impacts on air quality in rural communities.

## Data Availability

The data presented in this study are available from the corresponding author upon request. Other data such as meteorology and satellite data is available publicly with references in the text.

## Appendix

**Table 1.** Summary of previously reported δ^15^N-NO_x_ values sorted by emission source type.

Source type	δ^15^ N-NO_x_	Mean	Standard deviation	References
**Mobile source**	−8.1‰ to +9.8‰	−2.5‰	2.7‰	Walters, Goodwin, et al. (2015)*, Miller et al. (2017)
**Biomass burning**	−7‰ to +12‰	1.0‰	4.1‰	Fibiger & Hastings (2016)
**Stationary source**	−19.7‰ to −13.9‰	−16.5‰	1.7‰	Walters, Tharp, et al. (2015)
**Biogenic soil emission**	−59.8‰ to −14.2‰	−33.2‰	9.6‰	Li & Wang (2008), Yu & Elliot (2017), Miller et al. (2018)

* Only a portion of the measurements from this paper were used since they were measured with a cold, neutral engine, which wouldn’t be relevant for ambient temperatures.

**Table 2.** The 2022 California Emissions Projection Analysis Model (CEPAM) NO_x_ emission inventory and our estimated soil NO_x_ adjustments for Imperial County and the Coachella Valley, as well as for the combined SSAB, are shown. The CEPAM inventory is based on an annual average, so we averaged our soil source estimates for our sampling duration. Also shown are the adjustments to the NO_x_ inventory based on the average results from our field sampling campaign. The EPA’s National Emissions Inventory (NEI) for 2020 is also shown for comparison for Imperial County only.

Source Type	NEI (2020)	CEPAM (2022)			This Study		
	Imperial County (tons/d)	Imperial County (tons/d)	Coachella Valley (tons/d)	SSAB total (tons/d)	Imperial County (tons/d)	Coachella Valley (tons/d)	SSAB total (tons/d)
**Mobile**	11.9 (72.7%)	12.4 (81.8%)	15.9 (88.2%)	28.3 (85.2%)	12.4 (56.7%)	14.7 (69.9%)	28.2 (62.1%)
**Biomass Burning**	0.5 (3.1%)	0.1 (0.9%)	0.7 (4.0%)	0.8 (2.4%)	0.1 (0.6%)	0.6 (3.2%)	0.9 (1.9%)
**Stationary**	1.3 (8.0%)	1.7 (11.4%)	1.3 (7.4%)	3.0 (9.0%)	1.7 (7.9%)	1.4 (5.9%)	3.1 (6.7%)
**Biogenic Soil**	2.7 (16.2%)	0.9 (5.9%)	0.1 (0.4%)	1.0 (3.0%)	6.7 (34.7%)	4.7 (21.0%)	11.4 (29.2%)
**Total NO**_**x**_	**16.3**	**15.2**	**18.0**	**33.2**	**21.8**	**22.7**	**45.5**

**Table 3.** Field sampling results for δ^15^N-NO_2_ and estimated δ^15^N-NO_x_ based on isotopic fractionation calculations, as well as the results for δ^15^N-NO_3_ for Calipatria and Thermal.

	Calipatria			Thermal		
	δ^15^N-NO_2_	δ^15^N-NO_x_	δ^15^N-tNO_3_	δ^15^N-NO_2_	δ^15^N-NO_x_	δ^15^N-tNO_3_
**Jun 2022**	−13.2	−13.6	−0.02	No measurements		
**Jul 2022**	−13.4	−14.1	−2.6	−14.3	−16.1	−4.5
**Aug 2022**	−14.4	−14.9	−9.8	−4.6	−8.0	−4.8
**Sep 2022**	−15.6	−16.6	−3.4	−5.9	−8.0	N/A
**Oct 2022**	−5.3	−6.1	−1.1	−9.7	−12.0	−8.2
**Nov 2022**	−12.4	−13.4	−2.9	1.8	0.2	2.8
**Dec 2022**	−13.3	−14.9	−2.9	−7.5	−9.1	3.4
**Jan 2023**	−9.6	−11.6	−0.7	−1.5	−3.2	3.9
**Feb 2023**	−14.8	−15.5	0.1	−11.3	−12.0	2.6
**Apr 2023**	−16.0	−16.0	−3.4	−6.8	−7.6	1.8
**Average**	−12.8	−13.7	−2.7	−6.6	−8.4	−0.38

**Table 4.** Comparing our average soil NO_x_ flux estimate for Imperial County to CARB and other studies.

NOx flux	This study	CARB CEPAM	Oikawa et al. (2015)*	Almaraz et al. (2018)	Guo et al. (2020)	Luo et al. (2022)
**tons NO**_**x**_ **day**^**−1**^	6.7	0.9	17.2 – 111.2	140.0	1.6	13.5
**kg N ha**^**−1**^ **yr**^**−1**^	2.5	0.3	6.3 – 40.6	51.2	0.6	4.9

* Note that Oikawa et al., 2015 estimates were for September of 2012 only.
